# Impact of September 2019 Antarctic Sudden Stratospheric Warming on Mid‐Latitude Ionosphere and Thermosphere Over North America and Europe

**DOI:** 10.1029/2021GL094517

**Published:** 2021-07-30

**Authors:** Larisa P. Goncharenko, V. Lynn Harvey, Katelynn R. Greer, Shun‐Rong Zhang, Anthea J. Coster, Larry J. Paxton

**Affiliations:** ^1^ Massachusetts Institute of Technology Haystack Observatory Westford MA USA; ^2^ University of Colorado Laboratory for Atmospheric and Space Physics Boulder CO USA; ^3^ Applied Physics Laboratory John Hopkins University Laurel MD USA

**Keywords:** sudden stratospheric warming, ionosphere

## Abstract

Limited observational evidence indicates that ionospheric changes caused by Arctic sudden stratospheric warmings (SSWs) occur at middle latitudes in the Southern Hemisphere. However, it is not known if a similar interhemispheric linkage is produced by Antarctic SSWs. Here we examine thermospheric and ionospheric anomalies observed in September 2019 at middle latitudes in the Northern Hemisphere. We report persistent (at least 30 days) and strong (up to 80%–100%) positive anomalies in the daytime total electron content (TEC) and increases in the thermospheric O/N_2_ ratio in the western region of North America. However, central and eastern regions of North America experience moderate suppression of TEC reaching 20%–40% of the baseline. Different positive and negative anomalies are observed over the European sector. We hypothesize that regional differences in the TEC response could be related to modulation of thermospheric winds during SSWs, changes in thermospheric composition, and differences in declination angle.

## Key Points

- Large regional ionospheric anomalies at northern hemisphere middle latitudes are linked to the 2019 Antarctic sudden stratospheric warming
- The ionospheric response has a quasi‐semidiurnal behavior and is most likely driven by more than one mechanism
- We emphasize the potential role of thermospheric zonal wind and magnetic declination in the formation of these disturbances

## 1 Introduction

Significant progress in understanding mechanisms connecting the lower and upper atmosphere was achieved within the last decade by focusing on the analysis of sudden stratospheric warming events (SSWs). SSWs are large‐scale phenomena characterized by rapid temperature increases in the winter high‐latitude stratosphere, disruptions to the polar vortex, and decelerations or reversals of the stratospheric zonal winds. A review by Baldwin et al. (2021) summarizes the current state of knowledge about the mechanisms driving SSWs. Dramatic anomalies associated with SSWs are not limited to the stratosphere and have large impacts from the ocean to the ionosphere (see Pedatella et al., 2018 and references therein). Downward propagation of stratospheric disturbances affects tropospheric weather and leads to anomalous cold air outbreaks over Europe, Asia, and the eastern U.S. (Kolstad et al., 2010; King et al., 2019; Lehtonen & Karpechko, 2016). Dramatic changes are observed in the thermosphere and ionosphere where plasma temperatures can drop by ∼100–150K (L. Goncharenko & Zhang, 2008; Conde & Nicolls, 2010) and plasma density varies by up to a factor of 2 (Chau et al., 2010; L. P. Goncharenko et al., 2010; L. Goncharenko et al., 2013). Ionospheric and thermospheric anomalies related to SSWs are discussed in reviews by Chau et al. (2012) and L. Goncharenko et al. (2021).

One of the surprising features of ionospheric disturbances during Arctic SSWs is that the largest variations are seen not at the high or middle latitudes of the Northern Hemisphere (NH), but at low latitudes and middle latitudes of the Southern Hemisphere (SH). Low‐latitude variations have a semi‐diurnal character and reach their peaks near the crests of Equatorial Ionization Anomaly (EIA), located within 10–20° latitude of the magnetic equator. Both observational and modeling studies suggest that tropical variations are produced mainly through the vertical transport of plasma by enhanced electric field due to amplification of the E‐region dynamo mechanism (Fang et al., 2012; Pedatella & Liu, 2013). Ionospheric variations at the SH middle latitudes are much less understood. Fagundes et al. (2015) reported increases in total electron content (TEC) by almost a factor of 2 at a mid‐latitude location (Rio Grande: 53.8°S, 67.8°W), during the 2009 SSW. L. P. Goncharenko et al. (2018) saw similarly large increases in the daytime peak electron density *N*_*m*_ *F2* in Port Stanley (51.6°S, 57.9°W) ionosonde data during the 2013 SSW event. Analysis of TEC data along the 75°W longitude for the same event shows that nighttime decreases in TEC are largest near 40°S, and much weaker in the NH middle latitudes (L. P. Goncharenko et al., 2018). Pedatella and Maute (2015) noted larger ionospheric variations during two Arctic SSW events in the SH mid‐latitudes using both observations and simulations. They suggested that the mechanism responsible for these mid‐latitude disturbances is a modulation of thermospheric wind by an amplified semidiurnal lunar tide (Forbes & Zhang, 2012). However, simulations by Limpasuvan et al. (2016) indicated stronger amplification of semidiurnal tides in NH. This conclusion implies a stronger ionospheric response to Arctic SSWs in the NH, which contrasts with observational results discussed above.

SSW‐induced ionospheric anomalies in the middle latitudes of the opposite hemisphere and the mechanisms responsible for such anomalies are not fully understood. One of the reasons for this is the scarcity of observational data at SH middle latitudes. A record strong Antarctic SSW that occurred in September 2019 (Lim et al., 2020; Shen et al., 2020) enables addressing the question of inter‐hemispheric coupling during SSWs with unprecedented detail, as NH middle latitudes have exceptional coverage due to the dense networks of GNSS receivers. Studies of this SSW event demonstrate a record strong quasi 6‐days wave (Q6DW) in the mesosphere‐lower thermosphere (MLT) region and low‐latitude ionosphere (Yamazaki et al., 2020), multiple dynamo processes driving the Q6DW in the ionosphere (Lin et al., 2020), propagation of the Q6DW to 25–30°N in the Asian sector and a strong longitudinal variation in the ionospheric Q6DW at low latitudes (L. P. Goncharenko et al., 2020; Gu et al., 2021; Yamazaki et al., 2020). Other features include a Q10DW in the NH MLT (He et al., 2020), a Q2DW in TEC in the Asian sector at low latitudes, and quasi‐semidiurnal disturbances in TEC at low latitudes (L. P. Goncharenko et al., 2020). Here we utilize a variety of observational data to demonstrate that this Antarctic SSW produced anomalies in the middle‐latitude thermosphere and ionosphere in the NH and to suggest mechanisms responsible for these anomalies.

## 2 Results and Discussion

### 2.1 Stratospheric and Mesospheric Anomalies

**Figure 1:**
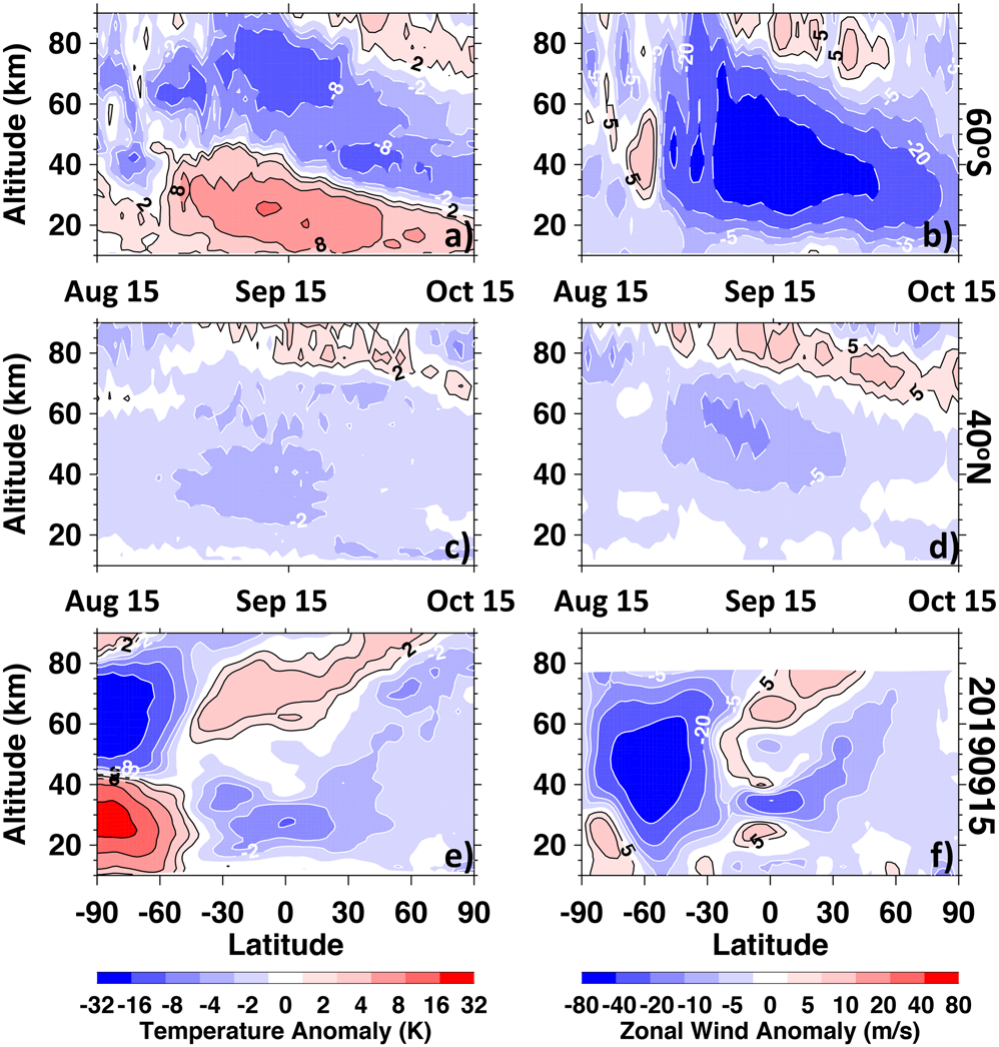
Altitude‐time sections of temperature and zonal wind anomalies in the middle atmosphere during the September 2019 Antarctic sudden stratospheric warmings event. Anomalies are computed by subtracting the 2004–2020 (except 2019) average from each day in 2019. (a) Temperature anomaly at 60°S. (b) Zonal mean zonal wind anomaly at 60°S. (c) Temperature anomaly at 40°N. (d) Zonal mean wind anomaly at 40°N. (e) Latitude‐height section of the temperature anomaly on September 15, 2019. (f) Latitude‐height section of the zonal wind anomaly on September 15, 2019. Panels (a)–(e) use Aura MLS data, panel (f) is based on MERRA2 data. Note the nonlinear color bar levels for both temperature and zonal wind anomalies.

Figure 1 illustrates spatio‐temporal variations in anomalies from the stratosphere to the MLT during the September 2019 SSW. These anomalies follow a well‐known “quad pattern”, when polar SH stratospheric warming and mesospheric cooling are accompanied by tropical stratospheric cooling and mesospheric warming (Limpasuvan et al., 2016; Randel, 1993). This quad pattern is associated with anomalous stratospheric and mesospheric circulation cells that result in adiabatic warming in the polar stratosphere and adiabatic cooling in the tropical stratosphere (and vice versa in the mesosphere). Figures 1a and 1b show the development of temperature and zonal wind anomalies, respectively, at 60°S. Positive stratospheric temperature anomalies at 60°S (values exceeding 8K, Figure 1a) persisted from late August to early October and peaked on 13 September. Mesospheric cooling between 60 and 80 km also reached its peak in mid‐September. Negative zonal wind anomalies at 60°S (values less than −20 m/s, Figure 1b) which are physically consistent with the temperature anomalies, persist from late August through mid‐October, and peak in mid‐September. Figures 1c and 1d demonstrate that long‐lasting middle atmospheric anomalies, while significantly weaker, are also observed at 40°N. Figure 1c shows SSW‐induced stratospheric cooling of 2–3 K at 40°N in mid‐September. Likewise, Figure 1d shows negative zonal wind anomalies in September, with values less than −5 m/s (a strengthening of the summer easterly jet) from 40–60 km. We note that the negative zonal wind anomaly reaches 9 m/s at 40°N and 50 km and represents a significant departure from the average background wind of −12 m/s (not shown here). The negative temperature (Figure 1c) and zonal wind (Figure 1d) anomalies are accompanied by positive anomalies near 80 km, as expected (e.g., Goldberg et al., 2004; Gumbel & Karlsson, 2011). Figure 1e further illustrates the global nature of the SSW‐induced temperature anomalies on September 15, 2019. In the extreme case of the September 2019 SSW, tropical temperature anomalies extend to the NH mid‐latitudes. Zonal wind anomalies (Figure 1f) also extend to middle and high latitudes of the NH, albeit they are weaker than in the SH. Pedatella and Liu (2013) showed that changes in the zonal mean middle atmospheric winds and temperatures are the primary drivers of the enhancement in the semidiurnal solar and lunar tides during SSWs, thus we can expect that during this Antarctic SSW event, semidiurnal tidal amplitudes are enhanced from SH high latitudes to NH mid‐latitudes.

### 2.2 Ionospheric Anomalies

For the purposes of this study, we used the CEDAR Madrigal TEC database and “quiet dynamic state” baseline (L. Goncharenko, 2021), as it provides a quantitative description of typical variability. The TEC data we used has a 1 × 1 degree resolution in latitude and longitude and 5 min resolution in time, and provides excellent coverage over continental U.S. and Europe (Vierinen et al., 2016). To clearly identify effects of the SSW and separate them from ionospheric variations due to changes in solar cycle, season, and geomagnetic activity, we tested two approaches for the description of background conditions. In the first approach, we followed the methodology originally described in L. P. Goncharenko et al. (2020) to develop the “quiet dynamic state” baseline for September low solar activity conditions (L. Goncharenko, 2021). We have also tested an alternative approach through the use of the empirical North America TEC model (Chen et al., 2015). We have concluded that these two approaches are consistent within 1.2–2.0 TECu average error estimates. Results of this study therefore do not depend on the selected method to describe background conditions.

**Figure 2:**
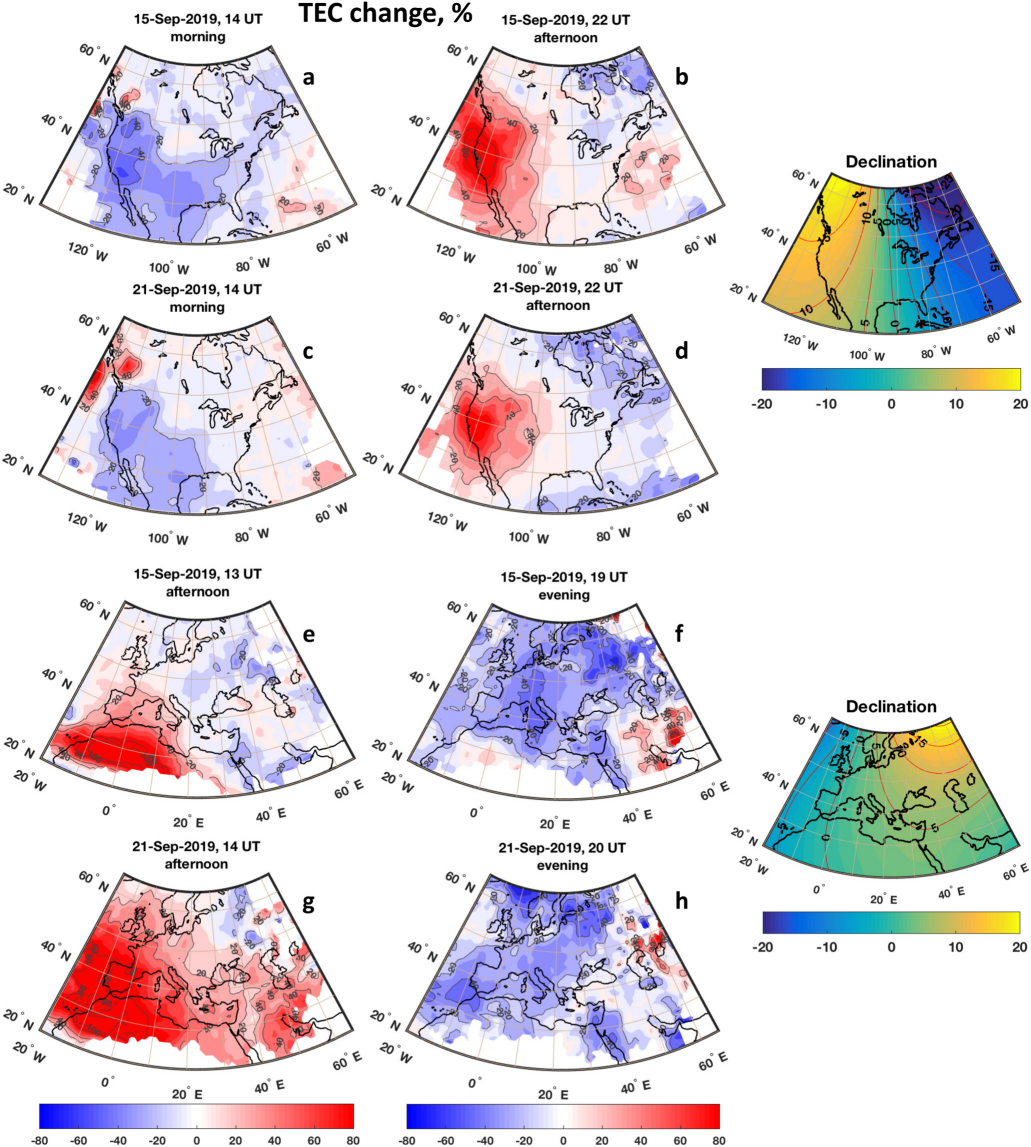
Ionospheric anomalies observed in September 2019. (a) and (b) Anomalies on September 15, 2019 over North America in the morning sector (14 UT) and afternoon sector (22 UT). (c) and (d) Same as (a)–(b), but for September 21, 2019. (e) and (f) Anomalies over North Africa and Europe on September 15, 2019 around noontime (13 UT) and in the evening (19 UT). (g) and (h) Same as (e)–(f), but for September 21, 2019. Inlets on the right side show variations in the magnetic declination angle over given regions.

This study focuses on anomalies over North America and Europe, where TEC data quality is highest. We hypothesize that the striking regional anomaly over the western U.S. is caused by an abatement or even reversal of the thermospheric zonal wind modified by the SSW, as zonal winds strongly influence ionospheric electron density in areas with larger magnetic declination angles (S.‐R. Zhang et al., 2011; 2012). Figure 2 illustrates the spatial development of ionospheric anomalies at NH middle latitudes. Figures 2a and 2b show TEC anomalies over North America on September 15, 2019 during morning (14 UT) and afternoon (22 UT) hours, respectively. Anomalies are expressed as a percentage change relative to the quiet time baseline. In the morning sector (Figure 2a), the prevailing change is a decrease of TEC by 20%–40%, with largest decreases over southern California (110–125°W, 30–50°N). In the afternoon sector (22UT, Figure 2b), the most striking feature is an increase up to 80%–100% in TEC observed at the same location. This positive daytime anomaly reaches its maximum at 40°N and is separated from low‐latitude anomalies observed near the crests of the EIA (L. P. Goncharenko et al., 2020). The central and eastern regions of North America experience only minor TEC variations, within the expected daily variability. The inlets on the right side of Figure 2 show declination angles (in degrees) over specific regions. The TEC variations observed over the western U.S. where declination angle exceeds 10° could be generated by an enhanced semidiurnal tide in the zonal wind, with more eastward wind in the morning (providing downward plasma motion and faster recombination) and more westward wind in the afternoon (providing upward plasma motion and slower recombination). The observed quasi‐semidiurnal variation in TEC is strongest in mid‐September, but is sustained for an extended period of time, as shown in Figures 2c and 2d for September 21, 2019.

The nature of TEC anomalies over Europe is very different, as illustrated in Figures 2e and 2f for September 15, 2019. Large positive anomalies, 100%–120% from the baseline, are seen in the early afternoon (13 UT) in a narrow longitudinal region (20°W–20°E) at 25–30°N, that is, near the northern crest of the EIA. Positive anomalies subside to 10%–20% poleward of 40°N. The magnetic equator is located at ∼10°N at these longitudes and ionospheric disturbances at the EIA crests are strongly linked to perturbations in vertical drifts and electric fields through the E‐region dynamo (Liu 2016). The positive disturbances over northwest Africa and western Europe are likely driven by perturbations in the electric field. The negative 20%–40% disturbances over Europe, which are observed several hours later at 19UT (Figure 2f), are not connected to the EIA and are seen over a broader range of latitudes, indicating contributions from mechanisms other than the electric field. Although TEC anomalies over Europe also exhibit quasi‐semidiurnal variations, similar to the quasi‐semidiurnal anomalies over North America, the local times of positive and negative variations are different, indicating contributions from different mechanisms.

As geomagnetic activity often increases during equinox (Lyatsky et al., 2001; Russell & McPherron, 1973), our observations of ionospheric anomalies related to this Antarctic SSW illustrate the need to include lower atmospheric forcing in ionosphere‐thermosphere studies to avoid misinterpretation of drivers and mechanisms, as suggested by Hagan et al. (2015) and Pedatella (2016). The largest positive TEC anomalies over Europe are seen on September 21, 2019 (Figure 2g), reaching higher latitudes and spanning a wider range of longitudes than on September 15. Negative TEC anomalies observed several hours later remained at the 20%–40% level (Figure 2h). As the SSW of September 2019 is associated with a record strong quasi‐6‐days wave in the middle atmosphere and low‐latitude ionosphere (L. P. Goncharenko et al., 2020; Gu et al., 2021; Lin et al., 2020; Miyoshi & Yamazaki, 2020; Yamazaki et al., 2020), the timing of the peak TEC anomalies on September 15, 2019 and September 21, 2019 indicates that they are linked to quasi‐6‐days oscillations that peak near the crests of EIA. Spectral analysis of TEC (not shown here) confirms the presence of oscillations with periods of 4–8 days in the extended latitudinal span in the European sector The regional nature of these disturbances could be related to secondary waves generated by the non‐linear interaction between the quasi‐6 days wave and the migrating semidiurnal tide (Miyoshi & Yamazaki, 2020). We note that both days, September 15 and 21, 2019 (as well as preceding days) were geomagnetically very quiet, with Kp = 1–3 and Kp = 1–2+. The magnitude of the TEC disturbances observed at middle latitudes during this SSW event is comparable to positive and negative effects (Buonsanto, 1999; Prolss, 1995) of geomagnetic storms.

**Figure 3:**
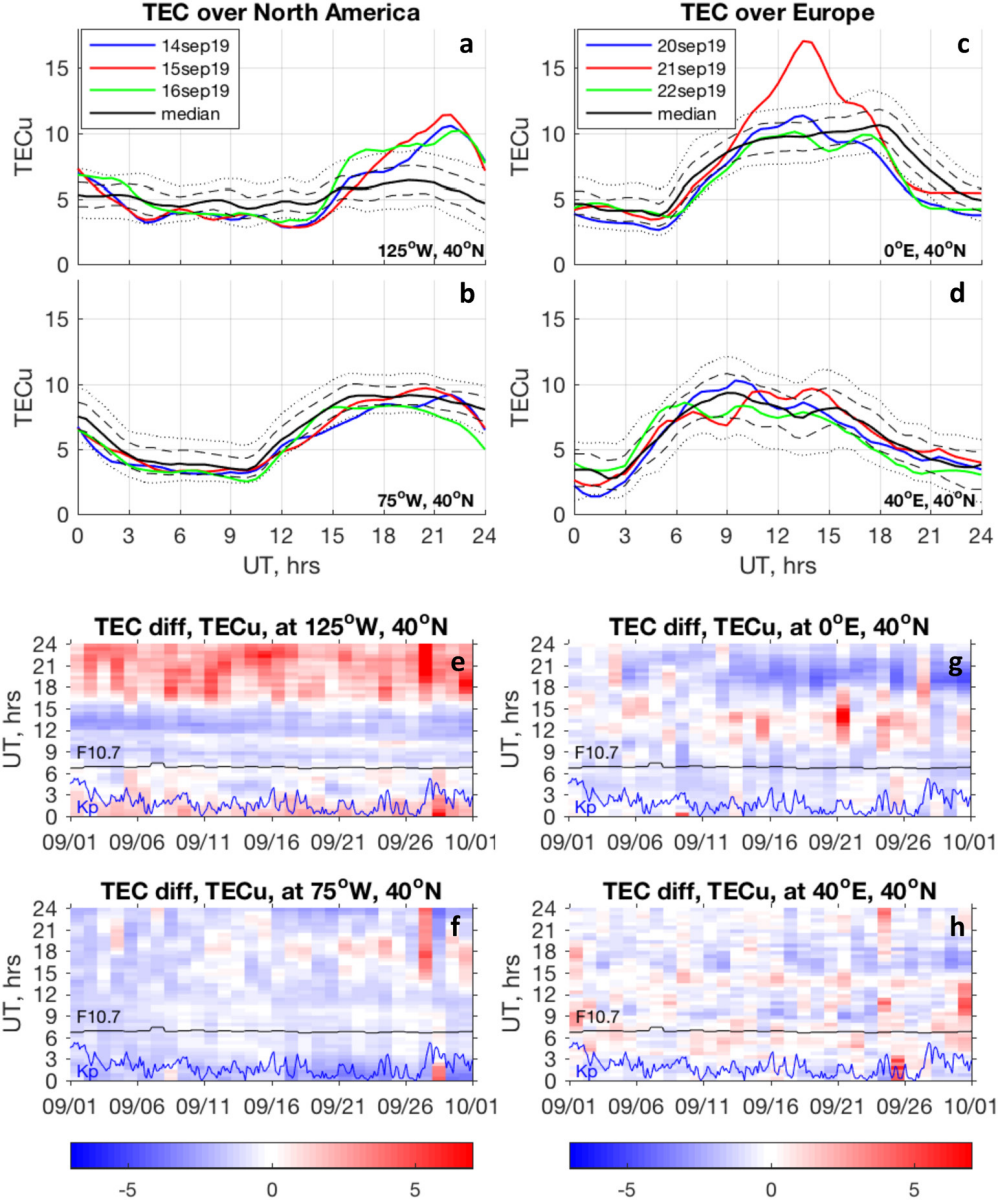
(Top part) TEC variations at 40°N at different longitudes in comparison with baseline. Panels (a–b) show variations over North America on September 14‐16, 2019. Panels (c–d) show variations over Europe on September 20–22, 2019. Thin dash lines show 25th and 75th percentiles and dotted lines show 10th and 90th percentiles to illustrate the level of typical quiet‐time variability. (Bottom part) Evolution of TEC anomalies in absolute units (TECu) in September 2019 at selected mid‐latitude locations over North America (panels e–f) and Europe (panels g–h). Variations in F10.7 index (divided by 10, black line) and Kp index (blue line) provide context for the TEC anomalies.

The differences in the baseline TEC behavior between the western and eastern U.S. are particularly large in our solar minimum condition case, and are likely to be enhanced by longitudinal differences in thermospheric zonal winds (K. Zhang et al., 2018). Strong daytime eastward winds seen by K. Zhang et al. (2018) at longitudes with positive magnetic declination (90–150°W) work to decrease electron density by moving ionospheric plasma to lower altitudes with higher recombination rates. Weakening or a reversal in this eastward zonal wind direction increases electron density and leads to higher TEC values. Observations of larger daytime TEC increases and larger nighttime decreases over the western U.S. in comparison with the eastern U.S. seen in Figures 2a–2d, 3a and 3b suggest that changes in the zonal wind during SSWs lead to the observed TEC behavior. As our ability to properly interpret TEC observations is hindered by the lack of observations of thermospheric winds, this hypothesis needs to be tested with model simulations and additional case studies. Figure 3 compares the baseline (median) with TEC observations during September 14–16, 2019 over North America (left panels, Figures 3a and 3b) and during September 20–22, 2019 over Europe (right panels, Figures 3c and 3d). Dramatic daytime (∼18–24 UT) increases in TEC in the western part of the U.S. (Figure 3a) exceed the 90th percentile of typical variability. TEC suppression is observed from nighttime to morning hours (∼3–15 UT), with negative anomalies often below tenth percentile. Maximum negative and positive variations occur several hours apart and exhibit a semidiurnal pattern. Similar disturbances are seen between 125 and 90°W (not shown), albeit with reduced strength. The TEC variations over the central and eastern U.S. have the same general character but smaller magnitudes and are mostly within expected quiet‐time variability.

Over the eastern U.S. (75°W, Figure 3b), the dominant signature is TEC suppression which is observed at most times of the day. We note large differences in the baseline TEC behavior (solid black line) between the western U.S. (125°W, Figure 3a) and the eastern U.S. (75°W, Figure 3b): daytime TEC is much larger over the eastern U.S. than over the western U.S., while nighttime TEC is higher over the western U.S. In other words, the ratio of the diurnal TEC maximum to the diurnal TEC minimum is larger over the eastern U.S. than over the western U.S. Such longitudinal differences in the baseline are produced by combined effects of thermospheric zonal winds and magnetic declination (S.‐R. Zhang et al., 2011, 2012) and are at least partially responsible for the regional nature of the observed TEC anomalies.

The TEC variations over Europe are illustrated in Figures 3c and 3d (right panels) for September 20–22, 2019, around the time of the largest disturbances shown in Figure 2g. A sharp daytime peak on September 21 to highly anomalous TEC values (30% higher than the 90th percentile) was accompanied by a strong decrease in the afternoon. Surrounding days experienced only minor daytime increases, within typical quiet time variations, and TEC suppression that dominated at most times of the day. Similar to observations over North America, significant positive TEC variations are seen only in a narrow longitudinal band; this signature subsides toward the east (at 20°E), and no obvious disturbances are seen at 40°E (Figure 3d) or 60°E (not shown).

The long‐lasting changes in TEC over the western U.S. and Europe are consistent with long‐lasting anomalies in the middle atmosphere shown in Figure 1 and imply that prolonged anomalies in the stratospheric wind system are imprinted on the thermosphere‐ionosphere system for an extended period of time. To illustrate the duration of the ionospheric anomalies, the bottom portion of Figure 3 presents the evolution of TEC anomalies over North America (panels 3e–3f) and over Europe (panels 3g–3h) for the entire month of September 2019 at the same locations as shown in the top portion of Figure 3. The most striking feature is that in the western U.S. (125°W, Figure 3e), positive TEC anomalies are observed for the entire month of September during the daytime (∼16–3 UT) while negative anomalies are observed at nighttime (∼6–15 UT). This behavior is seen only in a narrow longitude band (100–130°W) and subsides to the east of 100°W (not shown). Minimal TEC anomalies are seen over the central U.S. (90°W, not shown). The eastern U.S. (75°W, panel 3f) experiences moderate suppression of TEC for most local times (except minor increases during daytime, ∼17–19 UT). The TEC suppression becomes stronger, has a more pronounced quasi‐semidiurnal behavior, and extends for most of September over central Europe (0–20°E, Figure 3g), but subsides by 40°E (Figure 3h).

### 2.3 Thermospheric Anomalies

**Figure 4:**
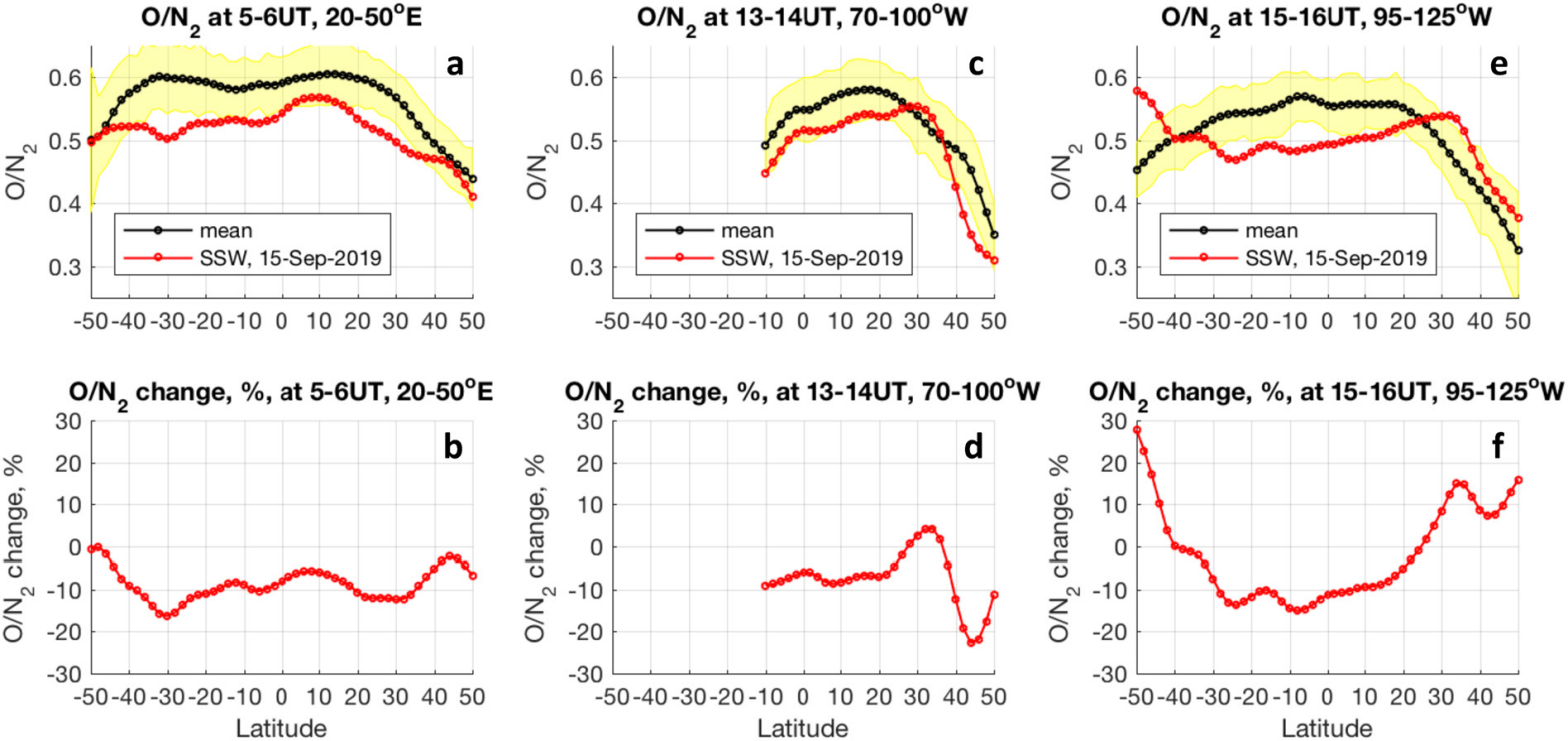
Evolution of thermospheric O/N2 at different longitudes. Top panels (a, c, e) compare O/N_2_ variations on September 15, 2019 (red) with background O/N_2_ behavior (black). Yellow shade indicates 1σ variation. Bottom panels (b, d, f) show differences in O/N_2_ between the background and the SSW day.

Numerous simulations have demonstrated that dissipation of upward propagating tides can affect vertical transport of atomic oxygen in the lower thermosphere that results in a reduction of thermospheric O/N_2_ ratio and, in turn, can contribute to a depletion in ionospheric electron density or TEC (Jones, Forbes & Hagan, 2014; Jones, Forbes, Hagan & Maute, 2014; Pedatella et al., 2016; Yamazaki & Richmond, 2013). Similarly, reduction in O/N_2_ ratio and electron density is also expected due to the dissipation of gravity waves (Jones et al., 2020) and planetary waves (Yue & Wang, 2014). We use observations of column O/N_2_ ratio ($\Sigma O/N_{2})\;$by the Global Ultraviolet Imager (GUVI) onboard the Thermosphere, Ionosphere, Mesosphere Energetics, and Dynamics (TIMED) satellite (Meier, 2021; Paxton et al., 2017, 2021; Strickland et al., 1995; Y. Zhang & Paxton, 2021) to examine whether anomalous $\Sigma O/N_{2}$ variations can be attributed to the SSW of September 2019. Background thermospheric $\Sigma O/N_{2}$ is calculated as a median value of all observations collected from 2008 to 2018, 2020 for low solar flux conditions (F10.7 < 80), low geomagnetic activity (Ap for the current day and previous day < 13), and centered on September 15 ± 10 days. Figure 4 compares background $\Sigma O/N_{2}$ (black) to $\Sigma O/N_{2}$ observations on September 15, 2019 (red) as a function of latitude as sampled along different orbits. The main difference between the background and SSW conditions seen at all longitude sectors is a ∼5%–15% depletion in $\Sigma O/N_{2}$ at low latitudes, ∼20°S to 20°N. This depletion is consistent with a 5%–10% $\Sigma O/N_{2}$ decrease in Global‐scale Observations of the Limb and Disk data reported by Oberheide et al. (2020) during the Arctic SSW in January 2019. It is also consistent with numerical simulations cited above, and is likely to be produced by the changes in the residual mean circulation related to the dissipation of SSW‐amplified tides and waves. This decrease in the low‐latitude $\Sigma O/N_{2}$ observation could contribute to a pronounced depletion of low‐latitude TEC (L. P. Goncharenko et al., 2020), as changes in $\Sigma O/N_{2}$ are well correlated with changes in TEC (Tsugawa et al., 2007; Y. Zhang & Paxton, 2021; Zhao et al., 2007). However, at middle latitudes (30–50°S and 30–50°N) the $\Sigma O/N_{2}$ change is more complex and depends on longitude. A decrease in $\Sigma O/N_{2}$ seen at middle latitudes over Europe (Figures 4a and 4b) and the central and eastern U.S. (Figures 4c and 4d) likely contributes to TEC depletions that dominate ionospheric responses at these locations (Figures 3b, 3c, 3d, 3f, 3g, and 3h). However, the $\Sigma O/N_{2}$ anomaly becomes positive at middle latitudes over the western U.S. (Figures 4e and 4f), where an increase in the daytime TEC is observed (Figures 2b, 3a and 3e), and thus can contribute to the formation of positive ionospheric changes over this region.

## 3 Conclusions

Ionospheric observations in September 2019 provide evidence of large (50%–100%) regional anomalies at middle latitudes of the northern hemisphere linked to the Antarctic sudden stratospheric warming. We expect that changes in thermospheric zonal wind play a major role in the formation of ionospheric anomalies, especially over western North America. The ionospheric response has a quasi‐semidiurnal behavior and is most likely driven by more than one mechanism, including variations in $\Sigma O/N_{2}$ induced by dissipation of waves (tides, gravity waves, planetary waves), variations in plasma drift due to the E‐region dynamo mechanism, and variations in thermospheric wind. This study emphasizes the potential role of thermospheric zonal wind and magnetic declination in the formation of regional ionospheric disturbances at middle latitudes, in addition to the role of secondary waves generated through the interaction of quasi‐6‐days waves and tides previously discussed in the literature. The strongest responses are limited to narrow longitudinal areas with widths of 20–40°, while other longitudes experience similar types of perturbations but with magnitudes within typical day‐to‐day variability. Positive and negative anomalies over the western U.S. (100–130°W) persist for the entire month of September. Over Europe (20°W to 20°E) the dominating response is long‐lasting TEC suppression superimposed on a quasi‐6‐days variation. Both positive and negative changes are observed in the thermospheric $\left(\Sigma O/N_{2}\right)$ ratio and can contribute to regional increases and suppressions of TEC.

The paper's additional key finding is that we cannot understand the variability and response of the thermosphere and ionosphere without understanding the episodic forcing from below and the extent of the persistent response of the system. Further, our ability to interpret ionospheric observations is limited by the uncertainty about the behavior of underlying thermospheric parameters, in particular the altitude profiles of winds, composition, and neutral density. The progress in understanding physical mechanisms responsible for the observed ionospheric behavior is hindered by the lack of thermospheric observations with appropriate spatio‐temporal resolution.

## Data Availability

The TEC data used as “quiet dynamic state” baseline in this study is available at Zenodo via http://doi.org/10.5281/zenodo.5083832. MLS v4.2 data are available from the NASA Goddard Space Flight Center for Earth Sciences Data and Information Services Center (DISC) at https://mls.jpl.nasa.gov/data/. MERRA‐2 data are available at https://gmao.gsfc.nasa.gov/reanalysis/. TIMED GUVI O/N2 data are available at http://guvitimed.jhuapl.edu/data_products.
